# Mediastinal myelolipoma showing gradual enlargement over 9 years: a case report

**DOI:** 10.1186/s13019-016-0482-3

**Published:** 2016-06-07

**Authors:** Tatsuaki Hosaka, Yoshinobu Hata, Takashi Makino, Hajime Otsuka, Satoshi Koezuka, Takashi Azumi, Kozue Ejima, Naobumi Tochigi, Kazutoshi Shibuya, Akira Iyoda

**Affiliations:** Division of Chest Surgery, Toho University School of Medicine, Tokyo, Japan; Department of Surgical Pathology, Toho University School of Medicine, Tokyo, Japan

**Keywords:** Mediastinal tumor, Myelolipoma, Video-assisted thoracoscopic surgery, Case report

## Abstract

**Background:**

Myelolipoma is a rare benign tumor composed of mature adipose tissue and normal hematopoietic tissue. Although surgical resection has been recommended due to the potential of progressive enlargement, the natural history of mediastinal myelolipoma has not yet been described. Herein we report a surgically resected mediastinal myelolipoma showing gradual enlargement over a period of 9 years.

**Case presentation:**

A 70-year-old woman presented with a posterior mediastinal mass shadow detected by computed tomography (CT) examination. She had a medical history of sigmoidectomy for colon cancer 13 years previously. A CT scan showed a smooth, well-demarcated 2.8 × 2.1-cm paravertebral mass shadow, composed of a fat density area and a soft tissue density area, which showed gradual enlargement from a 1.6 × 1.0-cm nodule 9 years previously. This was not accompanied by chronic anemia or hematologic disease including thalassemia, and no abnormal accumulation was observed on bone marrow scintigraphy or fluoro-2-deoxyglucose positron emission tomography. With a clinical diagnosis of mediastinal myelolipoma, surgical resection was performed, and pathological examination confirmed the diagnosis.

**Conclusions:**

We experienced a rare case with mediastinal myelolipoma showing gradual enlargement, with a tumor doubling time of 1,212 days.

## Background

Myelolipoma is a rare benign tumor composed of mature adipose tissue and normal hematopoietic tissue. Myelolipoma was first described by Gierke in 1905 and named by Oberling in 1929 [1]. Most myelolipomas are asymptomatic and discovered at autopsy, and their estimated incidence is 0.08 to 0.2 % [2]. The most common site of involvement is the adrenal gland, although extra-adrenal lesions have also been described, particularly in the presacral or retroperitoneal space [2]. Intrathoracic myelolipomas have rarely been reported [3–5]: 28 cases of mediastinal myelolipomas have been reviewed in the literature [5]. Although surgical resection has been recommended for mediastinal myelolipoma due to the potential of progressive enlargement, the natural history of mediastinal myelolipoma has not yet been described. Herein we report a surgically resected mediastinal myelolipoma showing gradual enlargement over a 9-year period.

## Case presentation

**Fig. 1:**
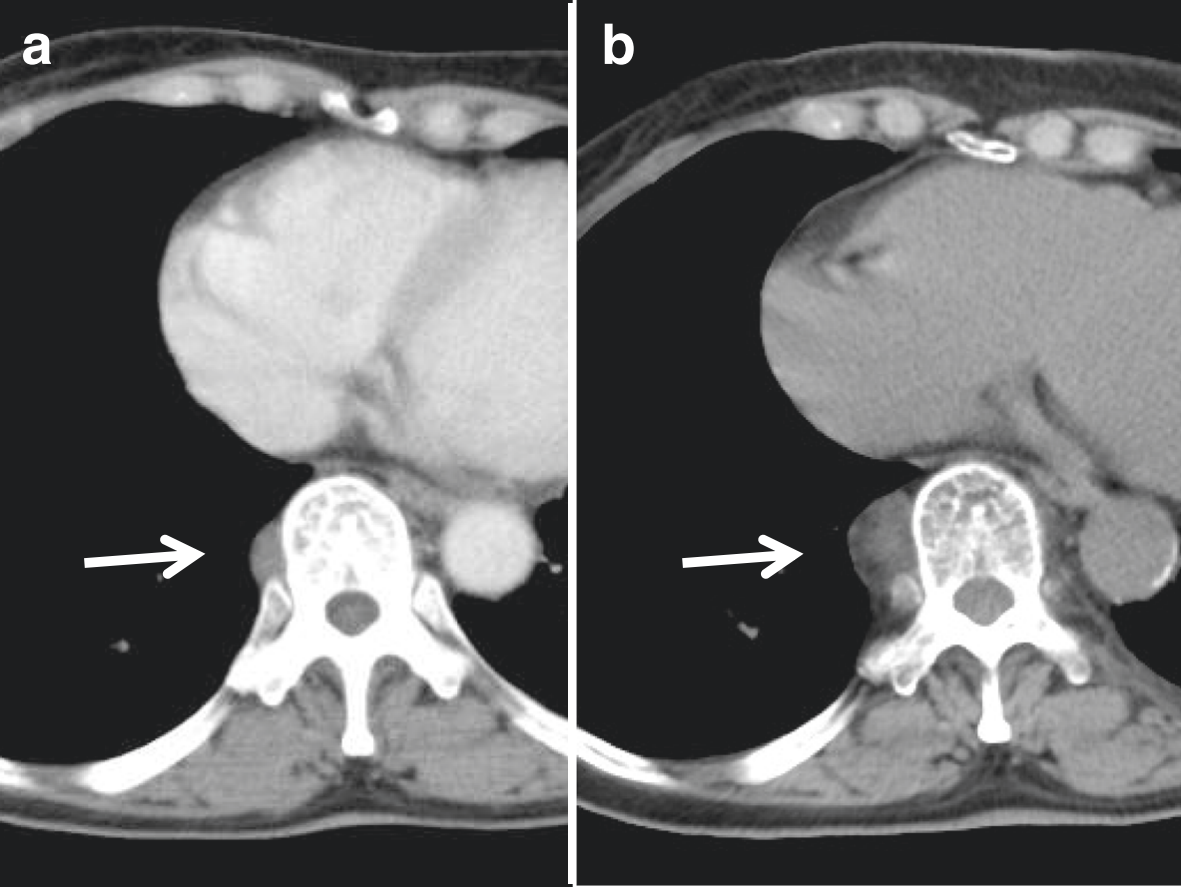
CT scan showing a 1.6 × 1.0-cm nodule 9 years previously (**a**), with gradual enlargement to a smooth, well-demarcated 2.8 × 2.1-cm paravertebral mass shadow, composed of a fat density area and a soft tissue density area (**b**)

**Fig. 2:**
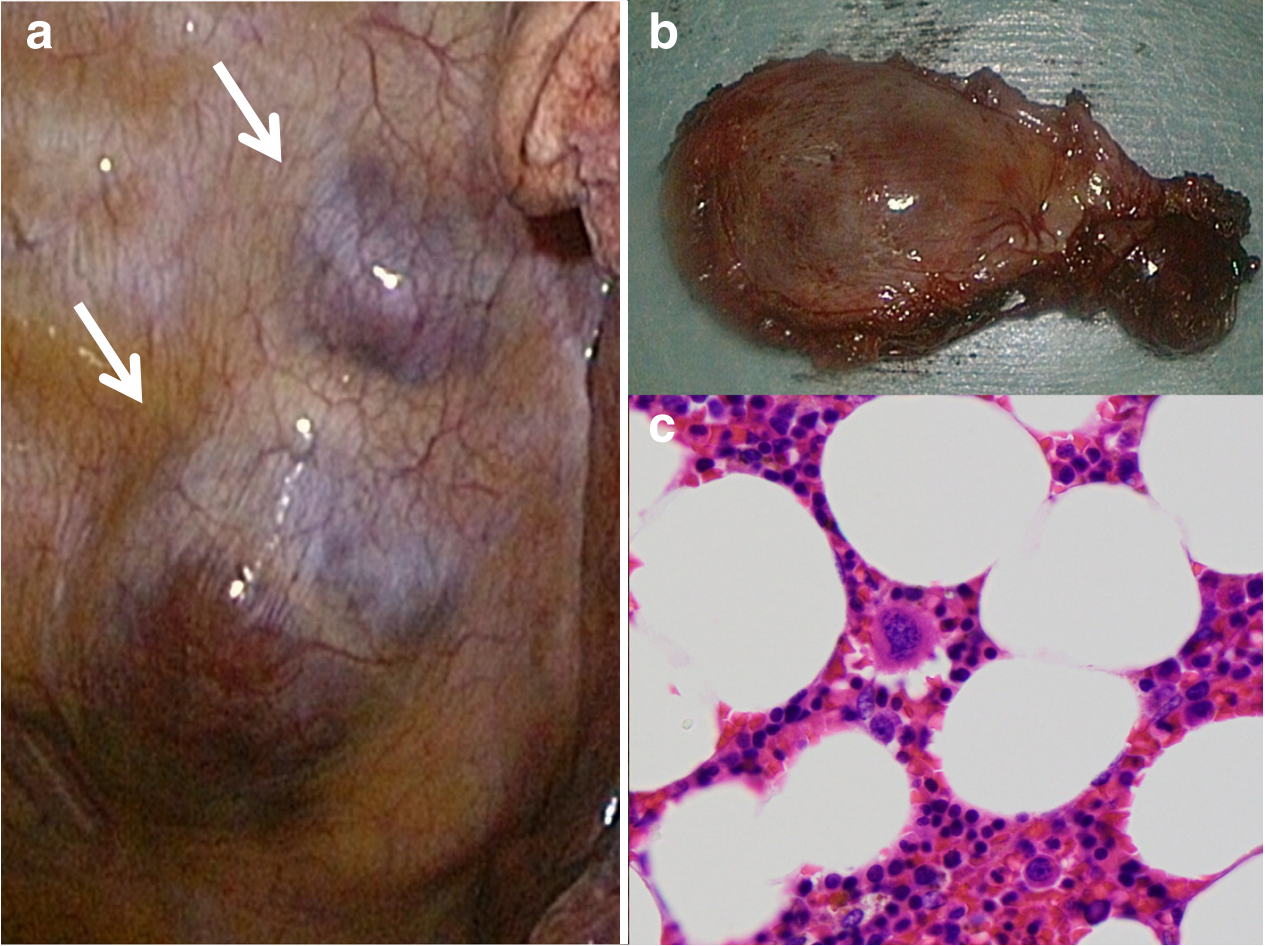
Video-assisted thoracoscopy showing a purple mass in the posterior mediastinum (**a**). Resected specimen showing a well-encapsulated soft tumor (**b**). Microscopic examination showing adipocytes admixed with normal hematopoietic elements, including megakaryocytes and cells of granulopoietic and erythropoietic lineages (H&E, x400, **c**)

A 70-year-old woman presented with a posterior mediastinal mass shadow detected by computed tomography (CT) examination triggered by a slightly elevated CEA level of 7.0 ng/mL. She had a medical history of sigmoidectomy for colon cancer 13 years previously and had a smoking history of 12 pack-years. A CT scan showed a smooth, well-demarcated 2.8 × 2.1-cm paravertebral mass shadow, composed of a fat density area and a soft tissue density area, which showed gradual enlargement from a 1.6 × 1.0-cm nodule 9 years previously (Fig. 1). Magnetic resonance imaging showed low signal intensity on T1- and T2-weighted images, with a small area of high T1 intensity that was depressed on fat depression imaging. Due to the existence of a fat component, mediastinal myelolipoma, extramedullary hematopoiesis, and well-differentiated liposarcoma were suspected. The mass was not accompanied by chronic anemia or hematologic disease including thalassemia, and no abnormal accumulation was observed on bone marrow scintigraphy or fluoro-2-deoxyglucose positron emission tomography. Based on a clinical diagnosis of mediastinal myelolipoma, surgical resection was performed with video-assisted thoracoscopic surgery. The lesion was a well-encapsulated, soft and purple mass in the posterior mediastinum (Fig. 2a, b). Frozen section examination was consistent with myelolipoma, and pathological examination confirmed the diagnosis (Fig. 2c). The postoperative course was uneventful, and the patient is well without recurrence 8 months after surgery.

## Discussion

Mediastinal myelolipomas are typically asymptomatic [3]. They tend to be incidentally detected during radiological investigation of unrelated symptoms. The tumor size ranges from 1.5 to 25 cm, suggesting the potential for continuous growth [5]. The surgical indication for mediastinal myelolipoma remains a matter of controversy. While myelolipoma is benign and generally asymptomatic, most myelolipomas are surgically removed due to the potential for progressive enlargement and uncertainty with respect to the preoperative diagnosis [4]. For early-stage disease, surgical resection with minimally invasive video-assisted thoracoscopic surgery is recommended, based on the assumption that delayed surgery might be associated with more surgical risks and invasiveness [4]. Although the potential for progressive growth has been repeatedly mentioned and surgical resection is recommended, the natural history of mediastinal myelolipoma has not yet been demonstrated, and the growth rate remains uncertain. With respect to intrathoracic myelolipoma, a case of pulmonary myelolipoma, showing slight enlargement from 1.8 cm to 2.5 cm over 13 years, has been reported [6]. The present case showed gradual enlargement from 1.6 × 1.0 cm to 2.8 × 2.1 cm over 9 years, with a tumor doubling time of 1,212 days. Although no conclusions can be drawn from these limited observations, these two cases of intrathoracic myelolipoma (mediastinal and pulmonary) demonstrated progressive enlargement as expected.

In contrast, not all cases of adrenal myelolipoma demonstrate continuous growth. The natural history of 16 adrenal myelolipomas has been reported to be an average of 3.2 years (range, 0.3 to 10.8 years) [7]. Among these, 13 tumors were serially imaged, with tumor size increasing in 6 tumors, decreasing in 2 tumors, and remaining unchanged in 5 tumors. Compared to the initial mean tumor size of 5.1 cm (range, 2.2 to 12 cm), the follow-up mean tumor size was 5.6 cm (range, 2.5 to 17 cm). These data may suggest that at least half of adrenal myelolipomas can be treated conservatively [8].

The other factor influencing indication for surgery is the risk of spontaneous rupture and bleeding, which has been reported to be related to tumor size [2, 8]. Among the 86 myelolipomas encountered at the Armed Forces Institute of Pathology, including 14 extra-adrenal myelolipomas, nine patients presented with acute hemorrhage [2]. Based on the observation that the smallest lesion that bled had a diameter of 8.5 cm while all of the others had a diameter > 10 cm (mean, 14.2 cm), elective surgery for large myelolipomas > 10 cm was recommended [2]. Another recommendation suggested that symptomatic tumors or myelolipomas > 7 cm should be surgically removed due to an increased risk of spontaneous rupture with retroperitoneal hemorrhage [8]. In contrast, small asymptomatic tumors < 4 cm can be monitored expectantly since they pose little risk of spontaneous rupture or bleeding [8].

Histologically, myelolipoma is a benign tumor composed of mature adipose tissue and hematopoietic cells, including myeloid, erythroid, and megakaryocytic elements, and occasionally lymphocytes [6]. Myelolipoma is distinct from extramedullary hematopoietic tissue, which occurs in association with hematologic disease and splenomegaly, does not contain fat, and is usually multifocal [2]. Extramedullary hematopoietic tumors show different characteristics with abnormal hematopoietic components, and erythroid hyperplasia is common [4]. CT scanning demonstrates an encapsulated mass, the density of which depends on the relative proportion of fat (low density) and hematopoietic tissue (high density); this may be enhanced following administration of intravenous contrast agents. Small amounts of calcification may also be observed [2, 6, 9]. CT-guided needle biopsy was reported to be useful for avoiding surgery in asymptomatic patients; however, the potential risks of dissemination in the case of malignant neoplasm and hemorrhage in the case of extramedullary hematopoiesis were noted [9].

## Conclusion

A surgical indication for mediastinal myelolipoma has been proposed due to the potential for continuous enlargement, uncertain preoperative diagnosis, and risk of spontaneous rupture and bleeding. While the natural history of mediastinal myelolipoma has not yet been described, the present case demonstrates gradual enlargement over a period of 9 years, with a tumor doubling time of 1,212 days.

## Abbreviation

CT: computed tomography
